# Tuberculous Osteomyelitis of the Hyoid Bone: A Case Report

**DOI:** 10.1155/2013/549564

**Published:** 2013-03-12

**Authors:** Paul Emerson, Ajay Philip, George M. Varghese, Regi Thomas

**Affiliations:** ^1^Department of ENT, UNIT-I, Christian Medical College, 632001 Vellore, India; ^2^Department of Medicine, Christian Medical College, 632001 Vellore, India

## Abstract

Skeletal tuberculosis is a well-known disease entity. We report the first case involving hyoid bone and the use of polymerase chain reaction-based test in detection and management. A 62-year-old male presented with neck swelling of a 20-day duration along with change of voice and dysphagia. Examination revealed a cystic, osteolytic lesion of the hyoid bone which histopathologically demonstrated features of granulomatous infection. A polymerase chain reaction test confirmed the diagnosis of tuberculosis.

## 1. Introduction

Tuberculosis (TB) is a chronic bacterial disease of the old and young alike affecting at least nine million of the world population annually of which one fifth are reported from India alone [1]. Extra pulmonary TB accounts for about 10 percent of these cases. Skeletal tuberculosis refers to involvement of the bones and/or joints and accounts for about 10% of the extra pulmonary cases. Tuberculosis osteomyelitis can occur in virtually any bone, including the ribs, skull, phalanx, pelvis, and long bones and is usually multimodal in origin. Spinal TB has been identified in the Egyptian mummies dating back to 9000 BC [2, 3]. Tuberculosis of bone and joints is most often secondary to lung contamination which spreads via haematogenous dissemination. The spine is most often affected, followed by the hips and the knees. Tuberculosis manifestations in the head and neck usually present as cervical lymphadenopathy followed by laryngeal involvement [4]; however involvement of the hyoid bone has not been reported so far in the literature. 

We report a case of isolated TB hyoid in view of the diagnostic dilemma posed by its presentation. An informed consent was taken from the patient.

## 2. Case Report

A 62-year-old man presented with history of a left-sided neck swelling for 20 days which was progressive in nature associated with odynophagia and hoarseness. He gave history of chronic nonproductive cough for three months with loss of appetite. There was no history of fever or hemoptysis. His past history was insignificant except for a residual left hemiparesis due to a cerebrovascular accident 7 years ago.

Examination revealed an ill-defined swelling on the left anterior aspect of the neck, approximately 4 × 3 cms which was cystic, fluctuant, and nontender. There were no palpable neck nodes, and oral cavity was normal.

Flexible nasal endoscopy (Olympus flexible endoscope) revealed a normal laryngopharynx except for a mucosal swelling on the left side of vallecula.

A provisional diagnosis of neck abscess was made. We aspirated the abscess with an 18G needle and drained 20 mL of pus. The pus was sent for routine culture, fungal smears, and AFB (acid fast bacilli) smears which did not yield any positive result.

**Figure 1:**
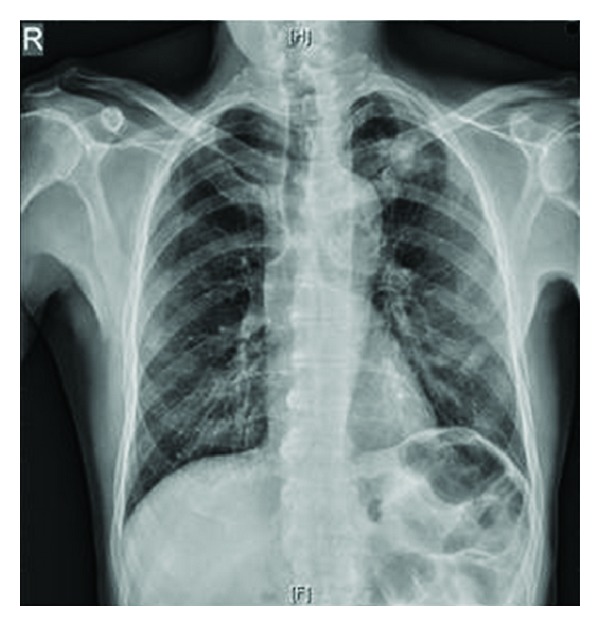
A cavity in the left upper zone of the left lung.

Three samples of sputum for AFB were sent in view of a cavity seen in the left upper zone on the chest X-ray (Figure 1), which were negative. Blood investigations done showed no immunocompromised status or other sources of infection.

**Figure 2:**
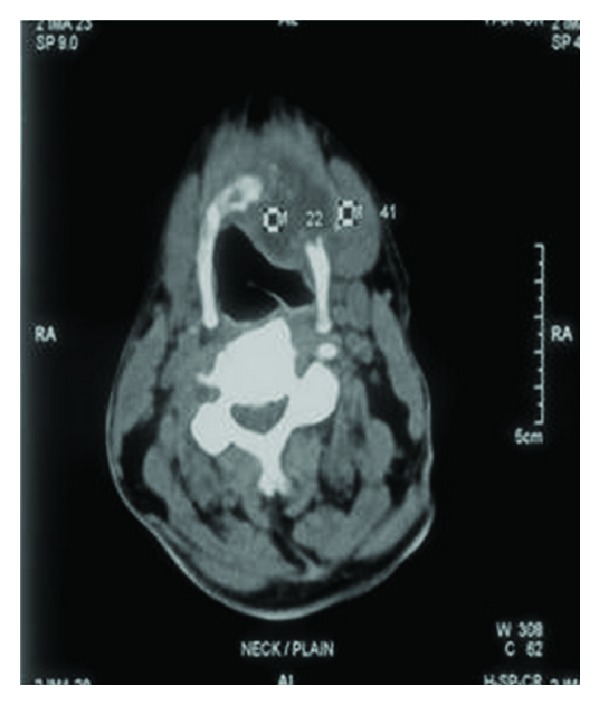
An ill-defined osteolytic lesion of the hyoid with peripherally enhancing collection.

A contrast enhanced computed tomography of the neck and thorax revealed an enhancing ill-defined osteolytic lesion of the body of hyoid with peripherally enhancing collection. The collection extended anteroinferiorly beneath the strap muscles along the anterior aspect of the thyroid cartilage (Figure 2).

**Figure 3:**
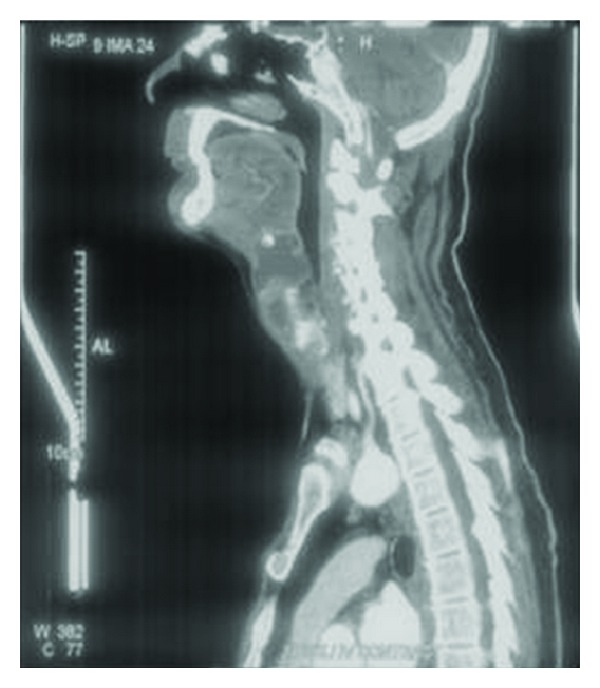
Involvement of the preepiglottic space.

Posteriorly the collection was found to protrude into the preepiglottic space (Figure 3). An incision and drainage of the abscess were planned. However as the patient suddenly developed a muffled voice, an elective tracheostomy was performed in anticipation of an impending airway compromise.

Intraoperatively there was a necrotic tissue with sero-sanguineous collection in the preepiglottic space. About 20 mL of fluid was drained. The body and the greater and lesser cornu of hyoid bone on the left side were found to be necrotic.

Histopathology of the hyoid revealed fibrocollagenous tissue with many discrete and confluent granulomas composed of epitheloid histiocytes and Langhans-type multinucleate giant cells rimmed by lymphocytes. Special stains done for fungal organisms were negative. A polymerase chain reaction study confirmed the diagnosis of tuberculosis.

## 3. Discussion

Tubercular osteomyelitis is a well-reported entity in the literature with involvement of the spine being the most common. The primary focus is in the lungs. It frequently presents as a “cold abscess” or as a swelling with modest erythema or pain and little or no local warmth. Active TB disease can develop immediately or after decades of latent infection. In nonendemic areas, musculoskeletal TB is commonly associated with late reactivation of infection and occurs mainly in adults. Involvement of hyoid bone has not yet been reported in the literature. Clinical diagnosis may be delayed due to the atypical presentation. Our study had no clinical features suggestive of TB except for the cavity noted in the chest radiography.

Culture tests are highly sensitive, but take 2–6 weeks to yield results and demand specialized materials to support the virulent mycobacteria in the culture. Sputum smear tests are quicker, producing results in about 30 minutes, but can only detect 15–75% of TB cases. Newer techniques of DNA polymerase chain reaction increase the chances of identifying and detecting tuberculosis infection quickly [5].

The new PCR-based TB diagnostic test called Xpert MTB/RIF is fast, sensitive, and automated [6]. It is a diagnostic test that can detect the presence of mycobacterium tuberculosis (MTB) and the resistance to rifampin (RIF), an antibiotic used to treat it. It is cost effective and highly sensitive in detection of tuberculosis and drug resistance.
